# Southern Dental Association

**Published:** 1887-12

**Authors:** 


AMERICAN JOURNAL

—OF—

DENTAL SCIENCE.

VOL. XXI. THIRD SERIES—DECEMBER, 1887. NO. 8.

ARTICLE I.

### SOUTHERN DENTAL ASSOCIATION, OLD POINT COMFORT, VA.—NINETEENTH ANNUAL SESSION.

REPORTED BY "MRS. M. W. J."

The Southern Dental Association convened in its 19th annual session at the Hygeia Hotel, Old Point Comfort, Va., Tuesday, August 30th, 1887.

The President, Dr. W. W. H. Thackston, occupying the chair, the first day was opened with prayer by the Rev. O. E. Herrick.

Circumstances having prevented the attendance of Governor Lee, who had been announced to deliver the address of welcome, Dr. Thackston made a brief address, which was responded to by Dr. J. H. Prewitt, of Kentucky, in an eloquent address on behalf of the Southern Dental Association.

Dr. V. E. Turner next welcomed all present in behalf of the Virginia Dental Society. This was followed by the annual address of the President.

The Association was called to order at 3:30 p. m. After some announcements from the Committee on Arrangements, the President introduced Prof. J. Taft, of Cincinnati, President of the Section of Dental and Oral Surgery of the International Medical Congress. Dr. Taft was suffering from a severe cold and made only a few remarks, cordially inviting all dentists present to participate in the proceedings of the Congress.

The President next introduced Prof. W. H. Morgan, of Vanderbilt University, Tenn., as one of the few *confreres* of his early days.

After the response of Dr. Morgan, Dr. George H. Torney, United States Army Surgeon, of Fortress Monroe, was next introduced (the Association was indebted to Dr. Torney for a corps of soldiers from the Fort as subjects for the clinics).

A number of visiting dentists from abroad, and from the North and West were introduced, and to whom the privileges of the floor were tendered.

Dr. Tandlak E. Sjolberg, of Stockholm, responded in behalf of the foreign members present.

Prof. J. B. Hodgkin, of Baltimore, offered the following resolutions:

WHEREAS, this Association has learned with deep regret of the death of Dr. J. R. Walker, of New Orleans, one of its oldest and most active members.

*Resolved*, That a committee of three be appointed to draft suitable resolutions, expressive of the sense of the loss this Association has sustained, and present them at a suitable time for consideration.

*Resolved*, That a memorial page be set apart in the roll book of this Association, to be inscribed with the names of the deceased members.

The resolutions were adopted and a committee appointed, with Dr. J. B. Hodgkin chairman.

Dr. E. A. Baldwin (Chicago) having prepared a paper for the Association, by special invitation, was assigned to the opening of the morning session, Wednesday.

The committees of scientific sections not being ready with reports on papers, adjourned to 10 a. m., Wednesday, August 31st.

WEDNESDAY, AUGUST 31—SECOND DAY.

First session called to order at 10 a. m., the President in the chair.

Dr. A. E. Baldwin, of Chicago, by resolution of the previous session was invited to the platform as the guest of the Association, and read a paper entitled "Immediate Root Filling."

The President desired to thank the author for the very able paper he has presented,

Dr. B. H. Catching (Atlanta, Ga.,) wished to ask Dr. Baldwin why he used 95 per cent. carbolic acid in preference to five per cent.

Dr. Baldwin replied that it was used simply in a state of diliquessence, in which there was 5 per cent. water. The 95 per cent. was used to distinguish it from the solid crystals. He had understood there were other papers bearing on the same subject, and regretted that they had not been read, that the subject, rather than his paper, might be discussed.

Dr. W. H. Morgan (Nashville, Tenn.) moved that if there were any other papers on the subject, they be read.

Dr. Catching suggested that if there were any voluntary papers on the subject not in the hands of the committee they be produced at once.

Dr. Winkler begged that they come up "like little men," and read them.

Prof. J. B. Hodgkin (Baltimore) said that he would like to stay at Old Point Comfort till Christmas, but it could not be. He moved that the subject be passed if there was nothing further to be said.

Dr. Marshall (Little Rock, Ark.,) read a paper entitled "Conservatism in Selecting Filling Materials."

Dr. Winkler said that the paper read by Dr. Baldwin was very valuable and its suggestions worthy of consideration, especially that relative to the thorough drying of the canal and the dessication of the tubuli. He did not agree with him in the use of the gutta-percha solution as root filling. He thought the best materials for root fillings were lead or carbolized wood, or gutta-percha points. He thought immediate root-filling limited to where the nerve was exposed and extirpated by driving in a wooden peg. In that case no treatment was needed, as all remains of the nerve and fibrous tissues would be immediately removed, the canal thoroughly cleaned and washed out, first with alcohol and then with carbolic acid, and wiped dry. When the canals were too small to admit the use of lead or wood, or gutta-percha points, he would use a mixture of iodoform and creosote in a stiff paste. Where dormant dead pulps were discovered, he cleaned the crown cavity thoroughly, opens the root canal and injects peroxide of hydrogen, letting it ooze out on a doylie until ebullition lessens and it finally comes out clear. Wiping it out thoroughly, fill with the iodoform and carbolic acid or creosote paste, pressing it down with a point of gutta-percha, but not sufficiently to force through the apex of the root. When old dead pulps were found, was afraid to fill immediately. Would dress them as above and wait at least one day. If they had been butchered with arsenic would wait longer.

Dr. J. C. Story (Dallas, Texas) said that to hear the brother from Chicago and the brother from Georgia talk, one might suppose that root canals were as big and as straight as gun barrels. But in Texas they were not made that way; they were crooked and small. That often it was impossible to get anything either in or out. He cleaned out root canals and he filled root canals as much as possible, but he did not fill them all. He uses oxychloride of zinc every time, and does not know that he has lost one. He uses iodoform because it outstinks all the odors of the tooth. He also uses oil of cloves and carbolic acid. If there is a blind abscess he *brings it to sight* by drilling through the alveolar process, and fills at once. He said he wanted the brethren to tell him more about those drills that have sense enough to go around curves and stop right at the end of the root; they had never got to Texas. He also wanted to know the ethology of pyorrhœa alveolaris. He felt that those two items would pay him for his long trip from Texas.

Dr.———(name not given by speaker) said that he had thought much on the subject of absolute dryness; that all were agreed as to the necessity for asepticism, but he did not think absolute permanent dryness could be attained, even with the hot air syringe; that moisture would find its way in through the tubuli. by capillary attraction.

Dr. C. E. Kells, Jr., (New Orleans) said that if the root filling was perfect there would be no danger from moisture; that the two could not occupy the same place at the same time. He said that Dr. Baldwin did not claim to fill *all roots*, neither did he (Kells). If a root canal was so fine that a nerve bristle could not enter, there was not enough nerve fibre to fear. If a drill could enter, then it should be filled. He uses a carbolized wooden peg, driven to the apex, and Guillois' oxychloride, but anything that would seal the apex would answer the purpose. If a child of seven or eight years of age breaks a tooth, say by a fall, before the root is developed, a peg of orange wood can be so shaped as to seal the open foramen. For five years he had done as he was taught at college, treating for weeks, but he had long since abandoned that useless practice. If an abscess followed root-filling, it could be as readily treated from the outside. He said that Dr. Winkler had used the terms carbolic acid and creosote as though they were synonymous, but that was not true. Carbolic acid would cauterize and leave an eschar, which was not the case of creosote.

Dr. J. Rollo Knapp (New Orleans) said that he would admit that he had failures, and they came back to him with abscesses. He did not think wood would always answer for a root filling; it would not do unless the canal was round. He would choose between wood or gold, or tin or gutta-percha points or pellets, or the solution of gutta-percha, according to the case. He also sometimes used oxychloride of zinc, or oxychloride and creosote—no one material was good in every case—sometimes he used the fluid gutta percha, and the solid to crowd it into the interstices. He used whatever he thought would be best and most effective for the case.

Dr. Winkler said he never used gold in root canals, but he admitted there were many that he would not fill at all.

Dr. Younger (of California) was introduced to the Association, and expressed his pleasure at meeting with "the Southern boys."

Dr. W. H. Morgan said that he had never listened to a better paper than the one read by Dr. Baldwin, but he must take issue with one or two points of minor importance. The trouble from lifeless teeth was the result of putrifaction—decomposition. If putrifaction could be prevented, it was immaterial whether or not the contents of the canals were removed. If thoroughly dessicated, decomposition could not take place, fluids being essential to the existence of germs. If the *cause* is removed we may safely trust to nature for the *cure*. If nature did not cure then we must resort to amputation, or trust to encysting. If the canals are enlarged so much as to remove a large portion of the dentinal tubuli with their semi-fluid contents, then there is less matter to decompose and putrify. This was his practicc. If the contents of the tubuli could be dessicated by hot air, then a great stride in advance had been made. He was, however, still skeptical on that point, and would continue to rely on antiseptics, though he would give the hot air system a fair trial also. He objected to the pumping in of liquid gutta-percha as liable to be forced through the apex, creating irritation in the soft tissues. In case of decomposed pulp with abscess, he would cut off the cause by thorough removal of contents and the use of disinfectants and antiseptics. It was stated incidentally that "the canals and tubuli were filled." He had never seen *tubuli* filled except by nature's own material, either normal or decomposed. Some fluid might possibly be forced in after thorough drying, but he had yet to see the tubuli *filled* by any process save nature's.

Dr. Baldwin thought he did not say *fill* the tubuli, but hermetically seal them.

Dr. Morgan said that sealing them at one end would not answer the purpose, since through anastomosis with the canaliculi of the cementum, fluids would still enter.

Dr. Geo. Eubank, (Birmingham, Ala.) to Dr. Morgan: Do you always devitalize exposed pulps, or do you endeavor to save them?

Dr. Morgan—I do not always destroy them.

Dr. T. Moore (Columbia, S. C.) did not think there were any drills that would go through all the contortions and contractions of some roots, but he did not deem it essential to fill all roots. He did not think the gutta-percha solution alone the best root filling, as from evaporation there would be some shrinkage and space left for other matter. After pumping in the liquid gutta-percha, he would drive in a point of gutta-percha, of lead, or of wood. In this way it could be filled so as to give no further trouble. He wished to ask Dr. Winkler if the process of driving out a nerve with the wooden peg was not very painful to the patient?

Dr. Winkler replied that until it was tried it was impossible to believe how entirely satisfactory this method was to both patient and operator, and how little sign of feeling was evinced by the patient.

Dr. W. H. Richards (Knoxville, Tenn.) referred to Dr. Baldwin's method of devitalizing and then waiting a week or ten days before making any attempt to remove the pulp. His own practice was to make the devitalizing application (arsenical paste) at say 8 a. m., and remove the pulp at 12

giving very little pain. At this period the congestion from the arsenic acted as an anæsthetic, and four hours after the application the nerve can be removed to the apex without pain. Later than that, inflammation sets in, and then suppuration if you wait a week or ten days. After removing the nerve the canals should be filled with a paste of 95 per cent. carbolic acid and iodoform for twenty-four hours, to prevent septic formations in the tubuli. He thought that if Dr. Morgan cut until he removed all the tubuli, he would have a very frail shell left. He did not see any necessity for drilling to the apex. He cleaned as best he could, but did not attempt to drill out; the small canals that could not be reached do not need filling. The little that is left in will do no harm if the door is so closed that no more can get in from the outside.

Dr. Tandlak E. Sjolberg (Stockholm, Sweden) said that the method of amputation of the pulp-bulb was much practiced in Europe. When the pulp was inflamed, (but not transformed to a shapeless mass) showing a new color not normal, if the root canals were very difficult, as in the mesial root of the inferior molar, they would cauterize with arsenical paste, letting this remain until next day; then opening the pulp cavity largely with a clean, sharp, new burr, they would remove the coronal portion of the pulp, really causing very little pain. Then clean out the pulp chamber thoroughly, checking the bleeding from the root, if any, by alum or what you choose. Place in the bottom of the cavity a small ball, the size of two or three pin heads, of carbolic acid or oxide of zinc, adapting it to the walls and covering it with a cap of platinum. Fill over this with cement, and finish with amalgam. This requires only two sittings, and is used where the canals cannot be cleaned and filled. Perfect cleanliness of the instruments is very important in this method, using a new burr and dipping it in carbolic acid.

Dr. McKellops (St. Louis) said that this subject was worn almost threadbare, we had been at it so long, and still there was the same want of success. No man could honestly say that he always makes a success. All who have ambition and pride try to do their work thoroughly. It is very well to say "devitalize the nerve and take it out." Sometimes it comes out very prettily with a few fibres of cotton on a broach; but again you may fuss for hours and defy any man to get it out. He said that he had hunted this country over and the Old world too, for the best broach; the finest made were the Swiss No. 10, but even with them he did not dare to go into all the buccal canals of molars. When does an abscess take place? What makes it form after we have done our very best, but not in so many other cases? He recently saw a patient in Chicago who had been treated for blind abscess. The teeth had been often filled. Finally the alveolar process had been taken off to reach it, and the septum taken out, $175 being charged for the operation, and yet when he saw him his face was perfectly immense. Dr. Baldwin said that we were safe with one or two remedies, but materia medica is a big field, and chemistry acts a big part. It may be microbes, it may be chemical action; the question is still open, hard students are at work on it. Men talk of their *successes*, but the abscessed roots that come to us for extraction with their oxychloride, and gutta-percha, and wooden pegs, etc., all in place, (some even filled with cotton, and smelling very sweetly!) tell another story than that of success. The man don't live who always makes a success of root filling. We sometimes think we had a success, when we learn afterwards that he went to some one else and had it out! These things come home to us all.

Dr. Beach (Clarksville, Tenn.)—Dr. Baldwin said in his paper that when he destroys a pulp he waits ten days for suppuration to take place. [A voice in the audience, "He said separation."]

Well! how will you have separation without suppuration? You will have decomposition of the entire pulp. My experience is that by waiting you have a little stump, perhaps ¼ or even 1-16 from the foramen that is more acutely sensitive than ever. I therefore use Dr. Winkler's method of stabbing with a peg of wood. It gives much less pain to the patient and is so promptly done. To wait ten days only increases the difficulty. I remove the day after making the application. Some one said there was nothing gained in drilling root canals. I think something is gained if the natural direction is followed and the canal rounded up; then you can fill with a round material smoothly rolled. I prefer a metallic filling to gutta-percha, which, in contact with moisture, becomes offensive, as is the case with wood also if not infiltrated with carbolic acid. From an experience of seventeen years with it, I think lead the best material. Even if it is driven through the epex it will be encysted, and not prove an irritant, so that you need not be afraid of it.

Dr. G. F. Evans (New York) said that he had an instrument for the application of dry heat in heating and drying dentine and root canals which he would exhibit at the clinics.

Dr. McKellops wished to recommend the gold broaches introduced by Dr. Herbst, made of platinum and gold, which if accidentally broken off could safely be left in as root filling, doing away with all dread of broken broaches.

Dr. Eubanks (Birmingham, Ala.) said that he found great advantages in drying root canals with alcohol, followed by chloroform, which acts well on the contents of the tubuli.

Dr. Stockton (New Jersey) said he was very glad he was not a young man coming to learn dentistry, for he should feel very much confused and puzzled, it was all so contradictory. One says dry out; another says you can't. One says drill them out; another says you can't because they are crooked, and another says shoot around the corners. The methods are as various as the men who advocate them. Many seem afraid to attack the pulp because it may hurt the patient. There is not as much pain in knocking it out as in poisoning it to death; not as much pain in knocking it in the head promptly as in putting in arsenic, whether for four hours or for ten days. What do you do when you expose a pulp?

Take it out! Better to have it between your fingers, than leave it to give inevitable future trouble. Tell the patient it will hurt a little. They will anticipate so much worse than they realize that they will say, "Is that all? I thought you were going to take the nerve out!" But I don't believe in immediate permanent fillings; I would fill temporarily, so that it can be readily reached and removed if trouble ensues.

I hope our dental manufacturers will yet give us some means of compressing air in heated reservoirs. They would confer a great benefit.

Dr. Allport (Chicago) said he wished to thank the members of the Southern Dental Association for the cordial reception they had met, and he wished to thank Dr. Baldwin for his excellent paper; when he undertakes to write, he goes to the bottom of his subject.

In the treatment of pulpless teeth, we must take into consideration their anatomy and their surroundings and connections. A tooth is not isolated; it has its vital connection with the rest of the system, and before it can be treated intelligently these must be understood. A tooth receives its vitality through the pulp. The cementum is nourished through the peridentium, but anastomosis between the canaliculi and tubuli is but very slight. Any circulation after the pulp is dead must come through the apical foramen. A living pulp must be either treated or destroyed; the dentine requires but little treatment. If the contents of the tubuli are so placed that they cannot decompose, it makes but little difference what the canal is filled with. A diseased pulp should be taken out, for it will die of itself, or from medical treatment or by surgery.

Heat affords the best treatment for the tubuli, it evaporates all the gases. A heated instrument is better than the hot air syringe—so hot that you can hear a sizzling sound. Then apply peroxide of hydrogen, expel anything that may have been left. Keep it up until no more gas is expelled then you can fill with safety. If the canal is thoroughly dried and purified, and the apex sealed it will be all right.

As the term, alveolar abscess is used, we would be led to believe that it came from gases evolved through the apex of the tooth within the alveolar. In nineteen cases out of twenty it is not an alveolar abscess at all; it is beyond the tooth, not around it. If gas escapes through the apex it follows the track of circulation. Where gases are formed within the tubuli of the tooth and pass through the cementum into the peridental membrane, we have an alveolar abscess, but this is not in one case out of a hundred. Gutta-percha, as a root filling, shrinks and becomes porous and offensive. Oxy-chloride of zinc has better chemical properties. Pump it in and then roll a little gold on a gold broach and force it gradually into the canals, coaxing it up and forcing the fluid laterally, finally withdrawing the broach but leaving the gold, leaving the root solidly filled to the apex. With this method I see no object in using wood.

Dr. McKellops said he was willing to go to Chicago only to see his young friend Allport get an instrument into a tooth hot enough to make it sizzle!

Dr. Allport said that the instrument was an instrument with a large bulb, to which was attached a broach. The bulb being heated very hot, the broach would convey sufficient heat to the chamber to make the contents almost boil, and would thoroughly dry the canals and all the dentine.

Dr. Morgan said that an identical instrument was used forty years ago to destroy nerves, burning them out with fire.

Dr. Beach moved that the subject close.

Dr. J. B. Hodgkin reminded Dr. Beach that Dr. Bald win had the right to close the dicussion by parliamentary usage.

Dr. Beach withdrew his motion.

Dr. Baldwin thanked the Association for the compliment of the lengthy discussion. He said that he always looked for more in the discussion than in the paper itself; that some of the speakers had failed to hear him, or had misunderstood his meaning. He had desired to state only his opinion, not an absolute certainty—man's work was never absolutely certain. We should never get too odd to learn, while the word of the youngest should be accorded the privilege of consideration. Some one said in the discussion, that he made it an invariable rule to devitalize, but judgment must be used! There can be no invariable rule used in anything.

In regard to waiting ten days, Dr. Baldwin said that he did so as a general rule, because the pulp was not a mass of nerve tissue only, but, also, of fibrous tissue, and it would be found less painful, when the line of demarkation between the living and the dead was formed. He did not say "one or two" remedies were sufficient. He said, and meant, "a few."

Dr. Freeman (Nashville, Tenn.) said that many of the best men present had not been heard from, and many who had spoken, had said only that which had been heard time out of mind. He hoped in future discussions they would try and bring out what was new, pungent and to the point. Lead for root fillings was one of the best things that could be used, and he hoped that those who had not yet used it would go home and try it, and report next year how they like it, and if they don't like it, tell why. It will follow along a tortuous canal and will flatten out if the roots are not round. He had not used arsenic for seventeen years, avoiding the troubles which others have with it. There are some pulps so tenacious of life that for weeks and weeks they will persist in living in spite of gouging and poisoning. Such nerves should be respected and allowed to live. If they want to die, let them die an easy, natural death, and then take them out.

Dr. Winkler—What do you use instead of arsenic?

Dr. Freeman—If I had a sick man I would not want to kill him! I want to cure him.

On motion the subject was passed and the Association adjourned to 3 p. m.

---

The Association was called to order again at 3 p. m., when, after the election of several new members, the subject of Operative Dentistry was continued, and Dr. Staples, of Sherman, Texas, read a paper entitled:

CAUSES OF FAILURES IN FILLINGS, followed by a voluntary essay from Dr. T. H. Parramore, Hampton, Va., on

SEPTIC SPONGE.

Another from Dr. Geo. H. Winkler, Augusta, Ga., on

SOFT GOLD FOILS.

and one from Dr. J. B. Hodgkin, of the Baltimore College, on

AMALGAMS, AND METHODS OF USING.

The general subject of Operative Dentistry, and especially the points raised in the papers read, were discussed until the hour for adjournment.

Among those taking part were Drs. Carr, Morgan, McKellops, Storey, Beach, and the authors of the different papers.

Dr. Morgan thought the old amalgams, as Townsend's original, were better than any of their so-called "improved." They turned the teeth black, but they also preserved the teeth, probably from the chemical action of the copper contained in the old coins used.

He did not consider any filling material equal to gold, though decay was always due to leakage, no matter what material was used. He also took issue with Dr. Stapler when he said that "dentists were born, not made." It was education that made the dentist. "Born dentists" were usually very poor specimens.

Dr. Storey came to the rescue of Texas, saying that all the education in the world could not make a dentist of a man unless the Lord intended it; that is, unless he had a natural aptitude for it. To educate meant to *draw out*, and unless there was something of the dentist *in* a man, it could not be drawn *out*.

Dr. McKellops asked how about John Hunter, and other early dentists, who had no means of obtaining a dental education?

Dr. Morgan replied that he was not there when they were around, and could not answer for them.

In reply to Dr. Winkler's paper, he said that much could be done with cohesive gold that no man could do with soft foil, and the very fact that the profession had so generally abandoned the use of soft foil, was sufficient proof that it was not the best.

Dr. Storey was struck with the new operation suggested by Dr. Parramore. He had never succeeded in capping nerves, but he would experiment on the new plan, even at the expense of his patients' feelings.

The discussion was lengthy and discursive, but nothing new was elicitcd, individual preferences *pro* and *con* being expressed at length, the usual theories propounded as to *why* gold was cohesive or non-cohesive, etc.; the general conclusion being, the best and wisest plan was to use either or both forms of gold, or amalgam, or cement, according to the nature of the case in hand, the health and age of the patient, the nature and location of the tooth and cavity, etc.

Professor Taft, president of the dental section of the Congress, addressed the members on the subject of the Congress, urging all to attend and participate in the proceedings.

At the close of the discussion adjourned to 8 p. m., Thursday, the day having been set aside for clinics.

Thursday, September 1st, was devoted to clinics, especially in operative dentistry and crown work.

Dr. Younger (San Francisco, Cal.) implanted an inferior central incisor for a soldier from Fortress Monroe. The tooth had been very loose and sore sometime before extraction with the fingers some months previous. Absorption was extensive, with apparently a very poor chance of drilling a new socket. The other central and the adjacent lateral had approached each other so much at the cutting edges that it was necessary to cut them away somewhat with a corundum disk to make space for the tooth to be implanted. The selected tooth having been sterilized and the root canal filled to the apex, the gum was dissected and the new socket formed with the triphine drills, the patient complaining less than is usually the case where a cavity is prepared for filling.

The other tooth implanted was a superior bicuspid. A flap of gum was dissected and turned back, to give access to the alveolus, and the socket drilled as before; the patient (a well-known dentist, Dr. Lester, of Virginia) stating that it was less painful than the preparation of a cavity for filling.

Dr. N. E. Baldwin illustrated the subject of his paper, in a case of immediate root-filling; a devitalized superior second bicuspid, of which Dr. B. S. Byrnes (Memphis, Tenn.) subsequently filled an approximate compound crown cavity. Dr. Byrnes also filled, with soft foil in ribbons, a buccal cavity in an inferior left third molar, working under difficulties; having only one broken excavator to work with, and using bibulous paper instead of rubber dam. Dr. Byrnes used, in these operations, the working model of his new automatic mallet, which can be readily attached to any hand piece by a simple attachment like putting a bit on, with a half-turn clamp. Every revolution of the engine gives a blow, a spring under the index finger controlling the strength of the blow, or checking it altogether. The force of the blow is given by a hammer, instead of by a powerful spring, thereby avoiding the unpleasant effect of a springing blow.

Dr. Wm. N. Morrison, of St. Louis, demonstrated his wonderful skill in filling tortuous roots with gold wire through a very small opening in the crown.

Dr. J. G. Morey proved the superior merits of his nerve drills. These drills are in the shape of a triangular reamer with a non-cutting point, with flexible shanks which conform to the curvatures of the roots. They are so constructed that there is no liability in using them of perforating either the nerve canal laterally, or the foramen. They are especially valuable in preparing root canals for the reception of pins in crowning or bridgework operations.

Dr. Morgan, of Staunton, Va., filled a labial cavity, demonstrating the advantages of Dr. D. B. Freeman's new doubleloop spring clamps, which hold the rubber dam in position over the tooth, crowd the gum back at the cervical border, and expose the cavity of decay in the centre of the oval loop.

Dr. Geo. Evans inserted one of his seamless gold contour crowns, made at the chair from an impression taken of the roots in Melotte's moldine; the crown made in a mold and burnished into form "a la Herbst." The crown was made and the roots prepared ready for cementing in twenty-five minutes.

Dr. Evans also exhibited his "bulb and broach" instruments for drying out all moisture from root canals and dentinal tubuli, as recommended Dr. Baldwin in his paper on root filling. The instrument consists of a large, oval mass of silver, which, being heated in the flame of the alcohol lamp, retains the heat a long time, and readily transmits it through a broach to the tooth substance. Dr. Evans exhibited specimens of his removable bridgework, removable porcelain fronts of teeth for crown and bridgework, etc.

Dr. Genese, of Baltimore President of the Maryland

State Dental Society, gave clinics in prosthetic dentistry, using his own articulator, upon which he modeled several cases consecutively. He made two complete upper dentures, using his new pinless teeth; one with plain and the other with gum-sections. He also demonstrated his method of packing without any excess of rubber, using Richel's automatic vulcanizer. By his methods his rubber plates have a highly finished surface, requiring no finishing, and also retain the natural contour of palate. Dr. Genese also demonstrated the use of his "syphon tongue-holder," and his "speculum and cheek distender."

Dr. L. P. Dotterer, of Charleston, S. C., restored a superior bicuspid with an all-gold crown made at the chair.

Dr. H. A. Parr, of New York City, restored to their original appearance and usefulness, two central incisors which had been broken off by a ball seven years ago. A plate had been worn over the roots, which were badly abscessed and buried under very unhealthy gums. The gums were dissected and pushed back, the roots cleaned, treated and filled to the apex. The roots were banded—one of them being split to the apex and porcelain front crowns, with pins running up to the roots, cemented into the bands, the whole operation being completed in about two hours.

Dr. Parr also applied his "universal separators" in four different patients, in each case obtaining sufficient space for thorough examination, or operations in less than three minutes.

The displays made by the dental manufacturing companies were very complete, not only in all the regular lines of goods, but in the number of new inventions and recent patents.

The S. S. White Company made their usual display of fine instruments, filling material, chairs, engines, and teeth of every style, shade and form, including their new "Bing's bridge teeth," having pins on the lateral surfaces for permanent attachment to adjoining teeth in the mouth.

Dr. N. T. Starr was busy striking up cap-crowns by their new die-plate and hub process, as described in the August *Cosmos*.

The "vulcan gold lining," for vulcanite plates, has the advantage of being cheaper than any metallic lining yet offered, costing only about eighty cents per full upper. This lining consists of gold and silver sweated together and rolled and hammered into foil of Nos. 20 and 40. The gold side is applied to the shellaced cast and burnished down. The silver being roughened by the sulphur of the rubber compound and adheres very tenaciously with no other preparation, aside from the usual mode of packing.

Among other things, in the S. S. White exhibit, may be mentioned the Mann vulcanizer; the battery, motor and switch-board of the Detroit Motor Co.; the Partz acid-gravity battery, which offers the great advantage of not requiring to be emptied for several months at a time, the zincs only needing to be cleaned twice or three times a year; the Shaw dental engine, an English patent, with a strong upright standard and double-grooved pulley, and a duplex driving spring, which gives five times the ordinary tension.

They have also an ingenious slip-joint connection with spring-cath for coupling hand pieces and right angles directly to dental engines.

In the Gideon Sibley exhibit was seen the Hood & Reynold's vulcanizer, William's separators, Brown's universal hand piece, and a fine assortment of teeth.

In the Welsh Dental Company's exhibit, the Richel self-packing, dry steam, automatic vulcanizer attracted much attention, and apparently possesses great merits. They exhibited a fine line of dental instruments and materials of all kinds, and are agents for Thompson's compound oxyhydrogen blow pipe, Dr. Genese's new pinless teeth, and other novelties.

They also had on sale the little book "Letters from a Mother to a Mother."

The American Dental Manufacturing Company exhibited a number of new patterns of fine instruments, and new filling materials.

Thursday having been devoted entirely to clinics, the association was called to order again at 7 p. m., the president in the chair, when Dr. P. Beadles (Danville, Va.,) read a voluntary essay, entitled:

DENTISTRY: THE OLD AND THE NEW.

Dr. Winkler then desired to reply briefly to some points raised in the discussion of his paper on soft foil. Statements has been made which he thought were in contradiction to known facts, and he did not wish them to go forth in the reports of the association unchallenged. He did not believe that the fumes of ammonia would make gold non-cohesive. He had been obliged to burn sulphur in a barrel and use the fumes to improve a lot of "sticky gold," when ammonia had failed to do what was claimed for it in that respect. Hope, the southern manufacturer of gold foils, had told him he added a small proportion of silver to make his gold non-cohesive.

(Quite a spirited exchange of *courtesies* here took place between Dr. McKellops and Dr. Winkler, regarding the clinic of the latter, which was subsequently settled *a l'aimable*.)

Dr. Storey (Texas) said that when he wanted his gold *extra sticky* he passed it through tincture of iodine, and then through the flame, and it would stick like North Carolina rosin.

Dr. R. Finley Hunt (Washington, D. C.) hoped, in the interests of science and nomenclature, the term *soft gold* might be discarded, for cohesive and non-cohesive foils were equally soft, and the terms cohesive and non-conesive were the only strictly correct terms to express the idea intended to be conveyed.

Dr. Morgan thought that Dr. Beadles claimed more for the progress made in scientific dentistry than the facts warrant. Any one who would go back and read papers published as far back as 1850, in the old American Journal, would see that we had made but little advance except in modifying modes of practice.

On motion, the subject of operative dentistry was passed.

Mechanical dentistry being called, Dr. J. R. Knapp offered a paper by Dr. Hilzim, (Mississippi,) which, on motion, was read by title:

MECHANICAL DENTISTRY, and left subject to the call of spoiration.

Dental Education called.

No paper presented.

Dental Hygiene being called next, Dr. W. D. Dunlap, (Selma, Ala.,) read a paper entitled :

DENTAL HYGIENE; A STUDY THAT BELONGS TO THE PEOPLE.

Dr. R. Finley Hunt (Washington) said that this was one of the most important subjects that could be brought to our attention. That this paper, like many others that had been read on the subject, while good and true as far as it goes, does not reach far enough. Dental Hygiene can not be limited to one point—as in *Cleanliness* as specified in the paper, or to the preparation of the cereals as expounded by Dr. John Allen, or to furnishing of adundance of limesalts to mothers and children, as held by our esteemed and lamented brother from New Orleans, Dr. J. R. Walker. The teeth may be kept as absolutely clean as possible—they may have had the most abundant supply of ingredients to make them, and to repair the waste of elements, and yet not be perfect health. It may be from lack of assimilation—or something else about the machine was not in proper condition—the teeth decayed in spite of every ease. Food-stuff in itself was not of so much importance as assimilation.

Examination of the skulls of our Northwestern Indians, of the Esquimaux and of the Sandwich Islanders, before they had acquired the habits and vices of civilization proves their teeth to have been remarkably good, with well-developed arches. Yet their food varied widely—that of the Esquimaux being highly carbonaceous—oil, blubber, etc., that of the Indians, game and the cereals; of the Sandwich Islandors largely vegetable products—and yet these very different forms of food produced equally good teeth.

Dental Hygiene cannot be confined to the mouth alone; the whole system must be prepared for the reception, preparation and assimilation of food stuffs. He hoped this would be made the subject of earnest study and investigation.

Dr. Beadles wished to ask Dr. Hunt if he would not include also the proper care of the mother during gestation?

Dr. Hunt replied that it was a matter of the greatest importance.

If there could be a return to natural conditious and habits of life our mothers would not need special prescriptions any more than did the Indians and Esquimaux mothers. The same plain food, well masticated, and free exercise in the open air and sunlight, and easy modes of dress, would induce a return to the type of well-developed arches and fine teeth found in our native tribes.

Dr. Wm. N. Morrison (St. Louis) said that much devolved upon the dentist of the future in this line. The best work that we can do falls into ruins for want of proper care. We should instruct our patients how to care for their teeth. His experience had been very different from that of Dr. Hunt as to the results of thorough cleanliness and care of the teeth. He believed that if a child were trained from infancy to keep the deciduous teeth well cleaned the second teeth would be all right. We have gone to extremes in the matter of prepared foods; we use too much acids and food that requires no mastication. If we were compelled to eat meat so tough that the jaws had to be assisted with the fists the teeth would be all the better for it. As we now live they are lost for want of use.

Dr. Storey said that in Texas they always had tough beef, it was proverbial, and in Texas, too, the teeth were as firm and strong as anywhere in the United States, except perhaps, Kentucky. In the "black-lands" of Texas, where there was a great deal of gypsum, there are heavy deposits of magnesia, lime and soda. The beautiful teeth spoken of, do not decay, but they are surrounded by tartar and loosen till they can be lifted out with the fingers.

In other portions of Texas, where the environments are different, the teeth are beautiful in appearance, but the enamel is very thin and the body of the tooth soft.

Dr. W. H. Morgan liked the trend of the paper, but thought it extravagant as to the scholastic population, and he thought the writer had better go over his figures again, even if "Uncle Sam" did get them up. He thought that in the study of Hygiene the object should be to attain the highest standard of health and the power to resist the encroachments of disease.

Dr. J. Y. Crawford (Nashville) said he did not wish to be regarded as fanatical, but he considered this subject one of the utmost importance to the profession, as a grand prophylaxy. The statistics quoted showed nineteen millions of school children; this alone shows the vast import of the subject. But a very small percentage have normal mouths, mouths in healthy condition, and fit to prepare food for the stomach. Dentistry is a grand prophylaxy, for by proper management of the teeth we ward off disease. If we could follow out the line indicated in the paper, with the enthusiasm of the writer, we would accomplish mnch for ourselves and much for the people. Our medical brethren smile derisively when we enlarge upon the importance of the care of the teeth, but it is none the less true that it is the most important hygienic question for the American people.

If our schools could be brought to require the same cleanliness of the teeth that they do of the face and hands, the same attention to the teeth that is demanded for the hair, we would get back to the perfect arches and the fine teeth of the aborigines; but that would not imply going back to barbarism! The subject is one of vast importance, and far reaching in its influence, even to those yet unborn. We owe it to those coming after us to confer lasting benefits on the human family.

Dr. Hunt expressed his satisfaction at finding so able an advocate of his position.

When, with all possible care and cleanliness, teeth are nevertheless lost, it is from lack of assimilation. The Indians and the Esquimaux, who carried their perfect teeth to the grave, had no tooth washes, but their systems were in perfect order.

We cannot expect to produce very marked results in the present generation, but can work for the future. As we improve the mothers of the day we improve the children of the future. The question of heredity enters largely into this matter. Good food, exercise, fresh air, sunlight, proper modes of diet, does not by any means imply a return to the squallor of barbarism, as remarked by one speaker.

To bring the human system to perfection of assimilation is the highest point of that science which affects the condition of the human race.

(*To be continued.*)

